# Oxidized phospholipids during microbial challenge: friend or foe?

**DOI:** 10.1038/s41435-024-00273-8

**Published:** 2024-04-22

**Authors:** Henrique G. Colaço, Kelsey Voss

**Affiliations:** 1 Institute of Hygiene and Applied Immunology, Center for Pathophysiology, Infectiology and Immunology, Medical University of Vienna, Vienna, Austria; 2 CeMM Research Center for Molecular Medicine of the Austrian Academy of Sciences, Vienna, Austria; 3 Department of Pathology, Microbiology and Immunology, Vanderbilt University Medical Center, Nashville, TN USA

**Keywords:** Sepsis, Antimicrobial responses

To fulfill their roles of surveillance and protecting the host from pathogens, phagocytes are faced with the challenging task of detecting invaders, distinguishing harmful from innocuous stimuli, and triggering the appropriate responses [1]. Sensing of pathogens occurs mainly through detection of microbial products or pathogen-associated molecular patterns (PAMPs) via host pattern recognition receptors (PRRs). PRRs encompass multiple families of receptors including the well-described Toll-like receptors (TLRs) that recognize a diverse range of conserved pathogen-derived ligands. However, commensal bacteria and microbes also possess these conserved microbial products, making the phagocytes’ job more complicated. Additionally, some PRRs detect host-derived damage-associated molecular patterns (DAMPs) produced by stressed, necrotic, or dead cells. The presence of both PAMPs and DAMPs can help phagocytes decide to mount an immune response, indicating both the presence of a pathogen and host cell damage presumably caused by that pathogen.

This “danger model” proposed by Polly Matzinger in the 1990s significantly advanced our understanding of immune cell decision-making in this context. Since then, however, the contributions of some PAMPs and DAMPs have expanded to have less straightforward roles. First described in plants, pathogen-derived effector molecules have evolved to initiate a protective immune response in the host. These effector proteins include toxins and virulence factors that promote infection and trigger a process known as “effector-triggered immunity” (ETI) [2]. In animals, ETI can include both virulence factors derived from the pathogen, or the host response to cellular damage [3]. Furthermore, activation of ETI can occur independently of PRRs, but it is unclear whether DAMPs can trigger ETI in eukaryotic cells and especially phagocytes.

The preprint from Di Gioia et al. [4] investigated the potential role of a host-derived DAMP, oxidized phospholipids (oxPLs) that result from non-enzymatic oxidation of phosphocholine-containing phospholipids. These oxPLs can be recognized by the innate immune system by a variety of PRRs and have established roles in driving atherosclerosis and non-alcoholic steatohepatitis, but their function in the context of infection is still disputed [5, 6]. Non-enzymatic oxidation generating oxPLs could occur due to general oxidative stress during inflammatory conditions. It is not clear in infections whether oxPLs are increased, and whether they promote or reduce inflammation.

Perhaps the best situation to test the potential contribution of oxPLs to inflammation and ETI during infection is sepsis. Sepsis is a dysregulated host response to infection where the delicate balance between pro- and anti-inflammatory mechanisms presents a critical challenge for therapeutic interventions [7]. Of note, sepsis also leads to extensive metabolic reprogramming with a consequent increase in circulating levels of numerous bioactive metabolites, which may trigger ETI and potentially affect the infection outcome.

Using several mouse models ranging from polymicrobial sepsis to exposure to a viral RNA analog, Di Gioia et al. [2] demonstrated that circulating levels of oxPLs are increased in acute inflammatory conditions. Accumulation of oxPLs was detected in blood and lymphoid organs such as the spleen, where they were particularly enriched in macrophages. In humans, oxPLs were increased in the bronchoalveolar lavage fluid of severe-to-critical SARS-CoV-2 patients, but not in patients with non-infectious inflammatory conditions such as sarcoidosis. Interestingly, repeated injections with a viral TLR ligand that mimics a persistent lung infection was sufficient to increase oxPLs in wild-type mice.

Using a transgenic mouse expressing an antibody that binds and neutralizes oxPLs (E06-scFv mice) [8], oxPLs were depleted to test their impact in vivo. In all infection models, oxPLs promoted worse morbidity and survival rates. Bone marrow-derived macrophages (BMDMs) treated with lipopolysaccharide (LPS) +/- oxPLs suggested that IL-10, a potent anti-inflammatory cytokine, was suppressed by oxPLs. Indeed, blockade of the IL-10 receptor was sufficient to abrogate the protective effect seen in E06-scFv mice. In support of this regulation, the authors found an inverse correlation between oxPL and IL-10 levels in pediatric patients with defined sepsis but not in critically ill patients without sepsis. Together, these data suggest that depletion of oxPLs improves survival by decreasing tissue damage associated with an excessive inflammatory response rather than by accelerating pathogen clearance.

To understand the action of oxPLs in IL-10 repression, the authors conducted a series of ex vivo experiments in phagocytes of mouse and human origin and concluded that oxPAPC (an oxPL) but not DPPC (a non-oxidized, biologically inert lipid) inhibits glycolysis and prevents the phosphorylation of AKT, both of which normally occur within minutes of exposure to LPS. Furthermore, inhibition of AKT activity with a small molecule (AKTi) recapitulated the effect of oxPLs in dampening IL-10 levels, indicating that AKT acts upstream of IL-10 and exerts a negative effect in its expression.

Once active, AKT regulates multiple inflammatory and metabolic processes through phosphorylation of its downstream targets. Thus, the question remained as to how oxPLs repress AKT function and decrease IL-10 expression. The activity of the AKT kinase is regulated by multiple upstream pathways, none of which were altered by oxPAPC in macrophages pre-treated with LPS. Nor were any known receptors of oxPAPC responsible for repressing AKT activity. Instead, oxPAPC was directly bound to AKT. Expression of truncated AKT variants narrowed down the binding domain for oxPAPC to the catalytic kinase domain and the effect was specific to oxPLs (not DPPC).

To further understand how AKT inhibition results in IL-10 repression, a metabolomics approach was performed in BMDMs exposed to LPS, oxPAPC, or AKTi. Intermediates of the methionine cycle such as S-adenosyl-homocysteine (SAH) were highly enriched in AKTi, LPS, and oxPAPC treated cells. Given that the conversion of S-adenosyl-methionine to SAH releases methyl groups that can be used for epigenetic modifications, the methyltransferase EZH2 was tested as an epigenetic regulator of the *Il10* locus. Indeed, oxPAPC treatment suppressed the phosphorylation of EZH2 induced by LPS and increased H3K27 trimethylation. Indeed, pharmacologically inhibiting EZH2 restored IL-10 secretion in vitro and increased survival in murine sepsis.

**Fig. 1:**
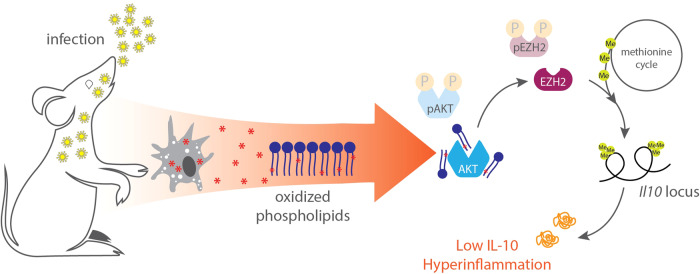
Oxidized phospholipids trigger a hyper-inflammatory state during infection. Infection triggers an accumulation of oxidized phospholipids (oxPLs) in macrophages which bind to AKT and subsequently alter the methylation status of the *Il10* gene. This epigenetic silencing of IL-10 production leads to a life-threatening dysregulation in inflammation.

Overall, this preprint showed that host-derived oxPLs are increased during microbial challenge in mice and humans, sequestering mainly within macrophages (Fig. 1). Although still unclear how macrophages “sense” these oxPLs, biochemical approaches demonstrated how these lipids can directly inhibit AKT activity, leading to a rewiring of metabolism and an increased pool of available methyl groups for histone modifications. This boosts EZH2 activity which ultimately silences *Il10* in macrophages. Intriguingly, the effects oxPLs in cellular metabolism, AKT inhibition, and IL-10 repression are maintained across a variety of stimuli (including PAMPS of bacterial, viral, and fungal origin) and are independent of the classical PRRs known to regulate cytokine expression. This is reminiscent of mechanisms of ETI initiated by microbial virulence factors, except that in this case the DAMP is host-derived and drives a hyper-inflammatory state independently of the type of pathogen or pathogen burden. Therefore, this study highlights a strong example of ETI in macrophages where oxPLs act as a host-derived DAMP. In severe infections such as sepsis, this ETI response drove excessive inflammation. However, in less critical situations it is possible that oxPLs turn from foe to friend and instead amplify the proper “danger” signals needed for a healthy immune response.
